# Acute study of the glycemic, insulinemic and triglyceride responses on white bread with peanuts and cashews in different forms (whole nut, nut butter and nut milk)

**DOI:** 10.3389/fnut.2026.1838715

**Published:** 2026-10-21

**Authors:** Saihah Mohamed Salleh, Sharifah Fatanah Bte Syed Abdullah, Kalpana Bhaskaran

**Affiliations:** Glycemic Index Research Unit, School of Applied Science, Temasek Polytechnic, Singapore, Singapore

**Keywords:** nut consumption, nut forms, postprandial glucose, postprandial insulin, postprandial triglyceride

## Abstract

Frequent nut consumption is associated with reduced cardiovascular disease (CVD) risk and mortality, particularly in individuals with diabetes, highlighting their potential health benefits. This study investigated the acute postprandial glycemic, insulinemic, and triglyceride responses to white bread consumed with peanuts and cashews in three forms: whole nuts, nut butter, and nut milk. Given that particle size and food form influence postprandial responses, it was hypothesized that nut milk would significantly modulate these metabolic parameters compared to whole nuts and nut butter. Fifteen healthy participants completed a randomized crossover study. Participants consumed a standardized serving of 42.5 g of peanuts or cashews in each form alongside two slices of white bread. Compared to white bread alone, all nut forms significantly attenuated postprandial glucose peaks and reduced glycemic responses, with a slight increase in insulin responses. Notably, nut butter and nut milk elicited 52 and 44% higher triglyceride responses, respectively, compared to whole nuts. Furthermore, peanuts resulted in a lower glucose incremental area under the curve (iAUC, 2 h) and a higher triglyceride response than cashews. However, these differences were not statistically significant. The incorporation of peanuts and cashews into white bread demonstrated improved glycemic control. While nut form primarily impacted triglyceride responses, suggesting a critical role for lipid bioaccessibility in modulating postprandial lipid metabolism, it did not significantly influence glucose or acute insulin responses. These findings suggest that prioritizing whole nuts over highly processed forms in a high-carbohydrate meal could be a beneficial dietary strategy for individuals managing diabetes or cardiovascular disease risk.

## 1 Introduction

The established association between frequent nut consumption and a decreased risk of cardiovascular disease (CVD) incidence and mortality is particularly relevant for individuals with diabetes. (1) The global burden of diabetes is substantial, with an estimated 463 million adults (aged 20–79 years) affected. The South-East Asia (SEA) region faces a significant challenge, with 107 million adults living with diabetes, indicating a regional prevalence of 13.2% in 2024, a rise from 8.8% in 2019. Critically, about one in two (42.7%) of those with diabetes in the SEA region are undiagnosed (2, 3).

Nuts are widely recognized for their substantial health benefits and are considered a highly nutritious addition to various diets. This wholesome food source is rich in healthy fats (monounsaturated and polyunsaturated fats), dietary fiber, vitamins, and minerals. Consistent consumption of nuts further contributes to positive health outcomes due to their abundance of bioactive compounds, including phytosterols and polyphenols (4, 5). Their regular intake has been linked to improved metabolic health, evidenced by reductions in total cholesterol and low-density lipoprotein (LDL) cholesterol levels, which in turn enhance insulin sensitivity and glycemic control (4, 5). Furthermore, nut consumption is associated with a reduced risk of CVD and may decrease the probability of developing type 2 diabetes (4, 5).

Recent research has increasingly focused on the acute postprandial effects of nuts consumption. Nuts are inherently low in available carbohydrates and elicit minimal direct postprandial glucose excursions when consumed alone. However, a recent randomized controlled crossover trial in overweight prediabetic Asian Chinese demonstrate that co-ingesting a nut-based matrix with white bread can suppress postprandial glucose response by up to 25%. This blunting effect is highly pronounced in both tree nuts and peanuts (6). Similarly, recent characterizations of distinct tree nuts like cashews confirm that their distinct macronutrient compositions offer practical dietary strategies for optimizing glycemic stability (7). However, while numerous studies have documented the health benefits of various nuts (8), research on the impact of different nut forms remains limited. Evidence suggests that particle size significantly influences digestion rates (9, 10), with variations in food forms (solid, semi-solid, and liquid) resulting in distinct *in vitro* digestion profiles and *in vivo* postprandial glucose and triglyceride responses (11).

This study aimed to determine the acute postprandial glycemic, insulinemic, and triglyceride responses to white bread consumed with peanuts (Arachis hypogaea) and cashews (Anacardium occidentale) in three forms: whole nuts, nut butter, and nut milk. White bread served as the carbohydrate control. Peanuts and cashews were selected due to their widespread consumption in Asia. To investigate the influence of food form on metabolic outcomes, nut milk (liquid), nut butter (semi-solid), and whole nuts (solid) were utilized. It was hypothesized that the liquid form (nut milk) would elicit a higher glycemic, insulinemic and triglyceride responses, compared to the solid (whole nuts) and semi-solid (nut butter) forms.

This hypothesis regarding the differential glycemic, insulinemic and triglyceride responses across nut forms may be attributed to the processing effects inherent in their preparation. The emulsification of fats during the conversion of nuts into liquid (nut milk) or semi-liquid (nut butter) forms results in smaller fat particles (12). These smaller particles facilitate more efficient absorption in the small intestine, potentially leading to a greater postprandial increase in triglycerides (12, 13). The enhanced bioavailability of lipids and subsequent elevation of triglyceride levels following fat emulsification in liquid foods is well-established (12, 13). Furthermore, liquid forms of food generally lead to more rapid gastric emptying, which typically results in faster carbohydrate absorption and thus a higher glycemic response (12, 13). In contrast, consumption of solid, whole nuts relies entirely on oral mastication, which results in incomplete structural breakdown and larger fractured particles. These larger particles preserve a degree of cellular encapsulation, thereby restricting lipid bioaccessibility compared to pre-emulsified states (13).

## 2 Methods

This study was conducted as an acute, non-therapeutic physiological investigation evaluating short-term postprandial metabolic responses to standard food items rather than a clinical trial evaluating health outcomes or therapeutic medical interventions. Consequently, registration in a clinical trial database was not required. However, this study received ethical approval from Temasek Polytechnic’s Institutional Review Board (IRB reference: IRB170102) prior to commencement. The study was conducted at the Glycemic Index Research Unit (GIRU) at Temasek Polytechnic, Singapore, with clinical data collection carried out between September 2018 and December 2018. All participants provided written informed consent after receiving a detailed explanation of the study protocol. Participants were recruited based on the following inclusion and exclusion criteria; Inclusion: Healthy males or non-pregnant females aged 19–60 years, absence of known food allergies or intolerances and no current use of medications known to affect glucose tolerance. Exclusion: Diagnosis of diabetes mellitus, use of anti-hyperglycemic drugs or insulin, major medical or surgical event requiring hospitalization within the preceding 3 months, presence of any disease or drug influencing nutrient digestion and absorption, and use of steroids, protease inhibitors, or antipsychotics. Habitual bread or nut consumption among the participants were not assessed. Anthropometric measurements were obtained using standardized methods in the fasting state on the morning of the first test. Height was measured to the nearest centimeter using a stadiometer (Seca Ltd., UK), with participants standing erect and barefoot. Body weight was recorded to the nearest 0.1 kg using the Seca 708 (Seca Ltd., UK), with participants barefoot. Body mass index (BMI) was calculated as weight (kg) divided by height (m)^2^. Participants with a BMI between 18.5 and 25 kg/m^2^ were included in the study. Eligibility was deliberately restricted to normal-weight participants to eliminate potential confounding variables associated with weight-related insulin resistance and to establish a consistent, baseline physiological response to the test foods.

Participants attended eight test sessions: two control sessions (white bread) and six experimental sessions, each consisting of white bread with peanuts or cashews in one of three forms (whole nuts, nut butter, or nut milk). The reference white bread control was administered in duplicate on two separate testing days, in accordance with standard ISO methodologies for glycemic response testing (ISO 26642:2010). Averaging the mean postprandial responses across both control sessions served to minimize intra-individual biological variability and establish a reliable reference baseline for iAUC calculations. The within-subject crossover design inherently enhances statistical power by minimizing inter-individual variation. In acute studies of carbohydrate metabolism, a cohort of 10 to 15 participants is well-established as sufficient to detect clinically meaningful physiological excursions across interconnected metabolic markers, including glucose, insulin, and triglycerides (14, 15). The reference white bread control sessions were administered at the start and conclusion of the study period. Meanwhile, the sequence of the six experimental nut treatment sessions was randomized using an automated online sequence generator^1^ in a counter-balanced crossover fashion to eliminate potential order, sequence, and carryover effects. All sessions were conducted in the morning, with a minimum two-day washout period between sessions. To ensure adequate carbohydrate intake and minimize baseline metabolic variation, participants were instructed to consume a balanced high-carbohydrate dinner (e.g., combining rice-, noodle-, or bread-based carbohydrates with protein and vegetables) and fast for 10–14 h overnight prior to each test session. Specific dietary examples and guidance to avoid protein-only meals were provided during the initial verbal study briefing, and participants were required to replicate the same meal pattern before every testing day. Participants were also asked to abstain from alcohol and vigorous physical activity for 24 h prior to each session. Immediately following the ingestion of each test meal, palatability ratings were measured using a 7-point hedonic scale (ranging from 1 = “Not Palatable” to 7 = “Very Palatable”).

### 2.1 Method preparation of test food

Table 1 details the preparation methods, serving sizes and nutritional composition for each test food. All experimental meals were standardized to contain 57 g of white bread (2 slices) paired with 42.5 g of the respective nut intervention (peanuts or cashews). Nut butters and nut milks were prepared using a food processor (Philips HR7772). For nut butters, 42.5 g of each nut was ground at Speed 3 until a visually smooth consistency was achieved. For nut milks, 42.5 g of each nut was blended with 120 mL of water at Speed 3 until visually smooth. The resulting nut milks were left unfiltered to ensure that all solid residue, fiber, and macronutrients were retained, maintaining nutritional equivalence across whole, butter, and milk formats. Due to the added water, total meal weights were 99.5 g for whole nut and nut butter treatments and 219.5 g or nut milk treatments.

**Table 1:** Method preparation and serving sizes and nutritional composition of the test food.

Code	Test food	Serving description	Energy (kcal)	Avail. CHO (g)	Fiber (g)	Protein (g)	Total fat (g)	Sat. fat (g)
WB	White bread (control 1 and 2)	*2 slices of white bread (57 g)*	150	31.2	1.4	5.6	1.0	0.5
WP	White bread + Whole peanuts	*2 slices (57 g) + 42.5 g peanuts*	409	35.8	4.2	17.4	22.5	4.3
PB	White bread + Peanut butter	*2 slices (57 g) + 42.5 g peanuts ground to butter*	409	35.8	4.2	17.4	22.5	4.3
PM	White bread + Peanut milk	*2 slices (57 g) + 42.5 g peanuts blended with 120 ml H_2_O*	409	35.8	4.2	17.4	22.5	4.3
WC	White bread + Whole cashews	*2 slices (57 g) + 42.5 g cashews*	404	41.5	3.3	14.2	20.8	4.4
CB	White bread + Cashew butter	*2 slices (57 g) + 42.5 g cashews ground to butter*	404	41.5	3.3	14.2	20.8	4.4
CM	White bread + Cashew milk	*2 slices (57 g) + 42.5 g cashews blended with 120 ml H_2_O*	404	41.5	3.3	14.2	20.8	4.4

Nutritional composition was determined by accredited third-party laboratory testing using standard analytical methods (Dumas method for protein, acid hydrolysis for fats/saturated fat, Prosky method for total dietary fiber, and calculation by difference for available carbohydrates).

Nutritional composition has been obtained from a third-party accredited food testing lab (Eurofins Food Integrity & Innovation Singapore) using standardized analytical methods. Total crude protein was quantified via the Dumas combustion method (using a nitrogen conversion factor of *N* x 6.25). Total fat was determined using the acid hydrolysis method, with individual fatty acid profiles quantified by gas chromatography and expressed as triglycerides. Total dietary fiber was measured using the enzymatic-gravimetric Prosky method, and sugar profiles (sucrose and total sugars) were analyzed by high-performance liquid chromatography (HPLC). Element concentrations (sodium) were quantified via Inductively Coupled Plasma Atomic Emission Spectrometry (ICP-OES/ICP-AES). Available carbohydrates were calculated by difference. Refer to Table 2 for the full detailed nutrient profile and composition.

**Table 2:** Nutrient profile of peanuts and cashews (based on 42.5 g serving size).

Nutrients	Peanuts (per 42.5 g)	Cashews (per 42.5 g)
Glycemic index		
Glycemic index value (17)*	13	25
Calories		
Calories (kcal)	259	254
Calories from fat		
Calories (kcal)	193	179
Fat by acid hydrolysis		
Fat (g)	21.5	19.8
Fatty acids calculated as triglycerides		
Saturated fatty acids (acid form) (g)	3.81	3.88
Monounsaturated fatty acids (acid form) (g)	11.77	11.43
Polyunsaturated fatty acids (acid form) (g)	4.93	3.22
Omega 6 fatty acids (g)	5.14	3.34
Omega 9 fatty acids (g)	12.20	11.82
Total fatty acids (g)	21.42	19.42
Protein (*N* x 6.25) Dumas method		
Protein (g)	11.8	8.6
Carbohydrates		
Total carbohydrates (%)	7.4	12.2
Available carbohydrates (%)	4.63	10.29
Total dietary fiber		
Total dietary fiber (g)	2.81	1.93
Sugar profile		
Sucrose (g)	1.57	2.55
Total sugar (g)	1.57	2.55
Elements by ICP emission spectrometry		
Sodium (mg)	<1.41^#^	3.26

Analytical methods used by the third-party testing laboratory: Protein via Dumas combustion (N x 6.25); Fat via acid hydrolysis; Sugar profile via HPLC; Sodium via ICP Emission Spectrometry. Available carbohydrate calculated by difference. #Sodium content was below the limit of detection (LOD <3.32mg/100g) and is reported as the calculated upper bound per serving (<1.41mg). *Values retrieved from The University of Sydney: GI Search (17).

Gardenia Enriched White Bread served as the control. For the experimental conditions, Planters Dry Roasted Peanuts and Nature’s Wonders Baked Nuts - Cashew were selected. These nuts were chosen for their unsalted composition and absence of additional ingredients, ensuring a focus on the inherent properties of the nuts. Their ready availability and consistent quality across local commercial brands in Singaporean supermarkets further support their relevance and practical implications for this study.

### 2.2 Blood collection and sampling

Capillary blood samples (~300 μL) were collected via finger-prick using Accu-Chek Safe T Pro Plus lancets (Roche Diabetes Care Asia Pte Ltd., Singapore). Fingertip capillary blood offers high sensitivity, comparable to arterialized venous blood sampling. Capillary sampling was chosen to minimize participant burden and the total blood volume collected throughout the 4-h study (14). Samples were dispensed into sodium fluoride/potassium oxalate tubes (~100 μL) for glucose analysis and serum separator tubes (~200 μL) for insulin and triglyceride analysis. Duplicate fasting blood samples were obtained at −5 and 0 min, and the mean value was used as the baseline. Participants consumed the control or test food (bread and nuts were consumed concurrently) within 12 min of the initial fasting blood draw. Subsequent blood samples were collected at 15, 30, 45, 60, 90, 120, 180, and 240 min post-ingestion. Participants remained seated throughout the 4-h testing period and were instructed to abstain from consuming additional food or beverages, engaging in significant physical activity, or excessive talking. Blood glucose was immediately analyzed using a calibrated YSI 2300 STAT Plus Glucose and Lactate Analyzer (YSI Incorporated, Yellow Springs, OH, United States). Serum separator tubes were allowed to clot for 30 min at room temperature, followed by centrifugation at 3,000 x g for 10 min. Serum samples were then analyzed for triglycerides at −5, 0, 30, 60, 120, 180, and 240 min using the cobas c111 analyzer (Roche Diagnostics; Material No. 04528778001) TRIGL Assay (TG GPO-PAP 200 T cobas c111, Material No. 04657594190). Insulin levels were determined at −5, 0, 15, 30, 45, 60, 90, 120, 180, and 240 min using an Amplified Luminescent Proximity Homogenous Assay human insulin detection assay (AlphaLISA® Human Insulin Immunoassay Kit, PerkinElmer Inc.; Catalogue No. AL3184C) and measured with an EnSpire® Multimode Plate Reader (PerkinElmer Inc.). The intra-assay and inter-assay coefficients of variation (CV%) were <2% for glucose (YSI 2300 STAT Plus); 3.3–9.7% and 12.3–12.9% for insulin (AlphaLISA®); and ≤3.1% for both intra- and inter-assay CV for triglycerides (cobas c111 TRIGL Assay). All analytical variations fell strictly within acceptable clinical laboratory thresholds, validating the precision of the datasets.

### 2.3 Analysis of test data

Following the Food and Agriculture Organization of the United Nations guidelines, the incremental area under the curve (iAUC) was calculated using the trapezoidal rule and a proprietary validated algorithm, disregarding the area below the fasting concentration. Data from the two duplicate white bread control sessions were averaged for each participant prior to statistical comparison and iAUC calculations, serving as the single reference control baseline. The iAUC for glucose, insulin, and triglycerides were determined for all control and test food. Glucose iAUC was calculated for two-, three-, and four-hour intervals. Evaluating 2-h iAUC aligns with international standards for acute glycemic response testing (ISO 26642:2010). The extended 3-h and 4-h monitoring windows were selected to accommodate the slower digestion and absorption kinetics of dietary lipids, where postprandial triglyceride peaks typically manifest between 180 and 240 min, as well as to observe late-phase insulin clearance and secondary metabolic stabilization. The Homeostasis Model Assessment for Insulin Resistance (HOMA-IR) was calculated for each visit using fasting glucose and insulin values according to the formula: (Fasting insulin (mIU/mL) x fasting glucose (mmol/L))/22.5 (16). Statistical analyses were performed using Microsoft Excel with Real Statistics Analysis ToolPak. All data are presented as mean ± standard error unless otherwise specified. A significance level of *α* = 0.05 was used for all statistical tests.

#### 2.3.1 Data pre-processing

Prior to statistical analysis, all raw data were carefully reviewed. Missing data points were identified and imputed using linear interpolation and Last Observation Carried Forward (LOCF). Outliers were identified using the three standard deviation rule, removed and subsequently treated as a missing data point. A total of two missing data points were handled by interpolation, and two missing final data points were handled by LOCF.

#### 2.3.2 Normality and homogeneity of variance assessment

Data distributions were assessed using the Shapiro–Wilk test, with supplementary confirmation by the D’Agostino-Pearson omnibus test where appropriate. Homogeneity of variance was assessed using Levene’s test. Glucose concentrations and glucose iAUC values demonstrated acceptable normality and homogeneity of variance and were therefore analyzed using the original untransformed data. Insulin data exhibited substantial departures from normality. Consequently, all insulin datasets were natural log-transformed (LN) prior to statistical analysis. Following transformation, normality and homogeneity of variance were considered acceptable for parametric analyses, and all subsequent insulin analyses were performed using LN-transformed values. Triglyceride data also demonstrated substantial non-normality. Natural log transformation improved the distribution of triglyceride iAUC values and these transformed data were subsequently analyzed using parametric methods. However, non-normality persisted in a substantial proportion of triglyceride concentration values collected at individual postprandial time points. Therefore, non-parametric statistical procedures were used for triglyceride concentration analyses over time, whereas LN-transformed triglyceride iAUC values were analyzed using parametric methods.

#### 2.3.3 Statistical tests

##### 2.3.3.1 Overall group comparisons (iAUC)

For glucose and LN-transformed insulin iAUC values, one-way analysis of variance (ANOVA) was performed to assess differences among the seven treatment conditions. When significant overall differences were observed, Tukey’s Honestly Significant Difference (HSD) test was used for *post hoc* pairwise comparisons. For LN-transformed triglyceride iAUC values, Welch’s ANOVA was employed when heterogeneity of variance remained present. *Post hoc* comparisons were conducted using the Games-Howell procedure where appropriate.

##### 2.3.3.2 Repeated measures over time

For glucose and LN-transformed insulin concentrations, two-factor analysis of variance was used to assess the effects of treatment condition, time, and their interaction across the postprandial period. When significant effects were identified, *post hoc* pairwise comparisons were performed and adjusted using the Holm-Bonferroni procedure to control the family-wise error rate. Because triglyceride concentrations remained non-normally distributed despite transformation, non-parametric analyses were used. Differences in triglyceride responses over time were evaluated using the Friedman test, followed by Nemenyi *post hoc* comparisons when appropriate.

##### 2.3.3.3 Specific pairwise comparisons

Where comparisons between individual treatment pairs were required at specific triglyceride time points, Mann–Whitney U tests were performed. *p*-values from multiple comparisons were adjusted using the Holm-Bonferroni procedure to control the family-wise error rate.

## 3 Results

### 3.1 Demographics information

A total of 15 healthy participants were enrolled and completed the study without any dropouts. The participants presented with a mean age of 34 ± 3.39 years and a mean body mass index (BMI) of 21.4 ± 0.43 kg/m^2^. The baseline characteristics of the study cohort are summarized in Table 3.

**Table 3:** Details of the participants involved in the study.

Subject ID	Gender	Ethnicity	Age	BMI (kg/m^2^)	HOMA-IR
060	F	Chinese	49	21.5	0.47
083	F	Malay	29	22.4	1.28
161	M	Chinese	33	21.9	1.73
165	F	Chinese	59	23.9	3.58
237	M	Chinese	42	19.0	0.87
239	F	Chinese	48	22.5	2.29
257	M	Chinese	23	21.9	1.46
262	F	Chinese	30	21.1	2.95
264	M	Chinese	56	24.2	1.84
277	F	Chinese	24	19.2	1.35
309	M	Chinese	23	22.9	0.93
311	M	Chinese	23	21.2	1.40
333	F	Malay	29	20.8	1.94
341	M	Chinese	23	18.9	0.74
349	M	Chinese	21	20.0	0.77
		Mean ± SE:	34 ± 3.39	21.4 ± 0.43	1.57 ± 0.22

### 3.2 Nutrient profile and composition of peanuts and cashews

The nutrient profile and composition of peanuts and cashews are presented in Table 2. The serving size is 1.5 oz., 42.5 grams based on the standard serving size by the U. S. Food and Drug Administration (USDA). The baseline nutritional composition of the test meals (detailed in Table 1) remained identical across all nut forms (whole nuts, butter, and milk).

Analytical methods used by the third-party testing laboratory: Protein via Dumas combustion (*N* x 6.25); Fat via acid hydrolysis; Sugar profile via HPLC; Sodium via ICP Emission Spectrometry. Available carbohydrate calculated by difference. *Values retrieved from The University of Sydney: GI Search (17).

Peanuts and cashews exhibit comparable caloric content, fat, saturated fatty acid, and monounsaturated fatty acid (MUFA) levels. However, cashews contain 64% more carbohydrates, 62% more total sugars, and 131% more sodium than peanuts. Conversely, peanuts contain 37% more protein and 46% more total dietary fiber than cashews. White bread demonstrates a higher carbohydrate content than both nut varieties, while the nuts possess substantially greater caloric density, protein, and fat. The present study evaluates realistic, practical serving combinations unlike traditional mechanistic studies that utilize strictly isoenergetic or macronutrient-matched test meals. By testing standard, commercially relevant servings, these findings aim to offer direct, practical relevance to everyday dietary guidelines.

### 3.3 Glycemic, insulinemic and triglyceride response and iAUC of the white bread (control) and white bread with peanuts and cashews in different forms

The glycemic, insulinemic and triglyceride response with 95% confidence intervals of White Bread (control) and White Bread with Peanuts and Cashews in different forms are as shown in Figure 1.

**Figure 1:**
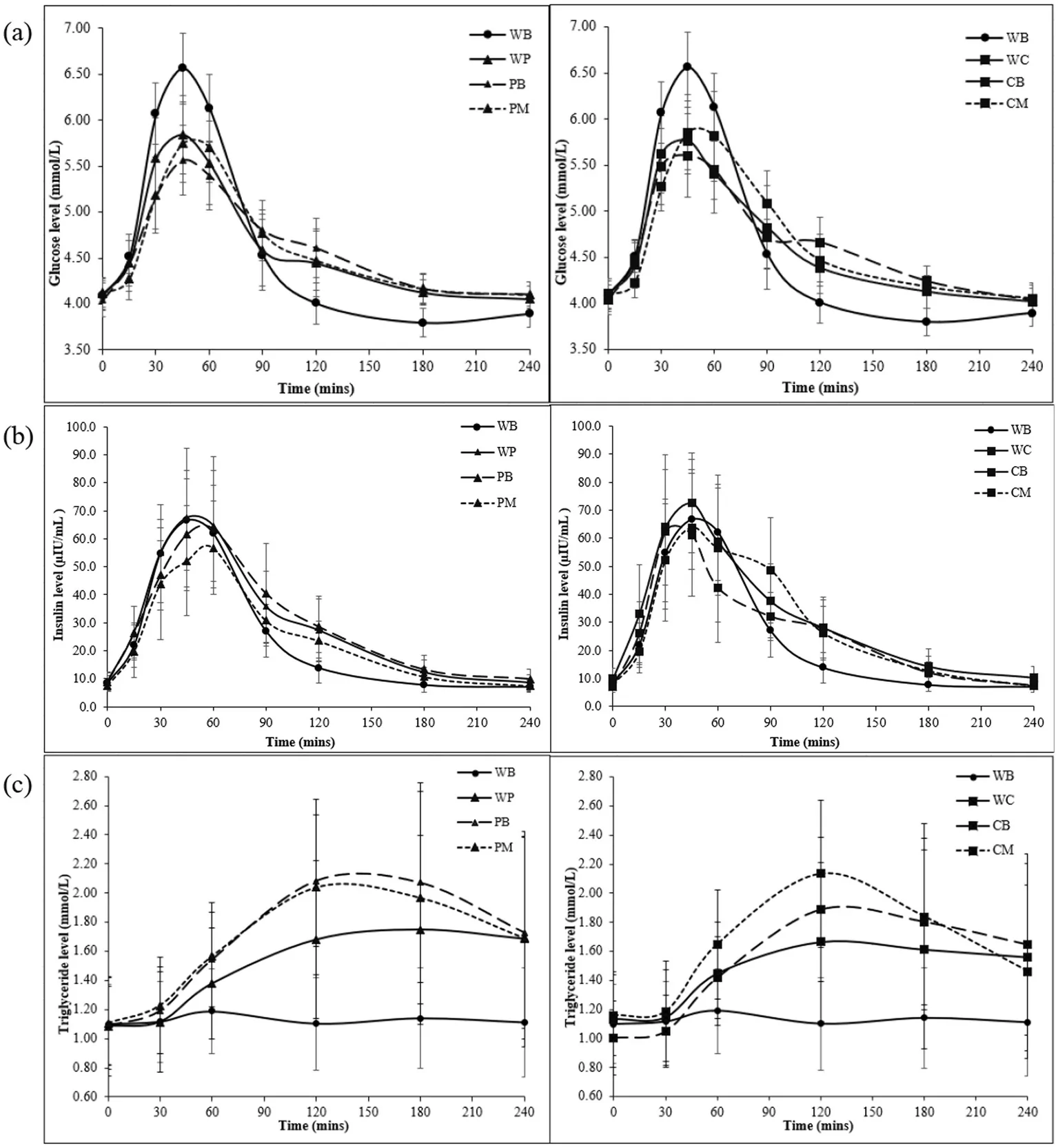
Mean postprandial responses for **(a)** glucose, **(b)** insulin, and **(c)** triglycerides following consumption of white bread alone or white bread with peanuts or cashews in different forms. WB, white bread control; WP, white bread with whole peanuts; WC, white bread with whole cashews; PB, white bread with peanut butter; CB, white bread with cashew butter; PM, white bread with peanut milk; CM, white bread with cashew milk. Data are presented as mean values with 95% confidence intervals. The WB curve represents the mean of duplicate reference white bread sessions conducted for each participant.

#### 3.3.1 Glucose

The white bread control (WB) elicited a rapid and substantial increase in blood glucose within the first 45 min, resulting in a high overall glycemic response. Glucose levels decreased below the fasting baseline at 120 min, followed by a modest rebound after 180 min. All test foods demonstrated a significantly lower postprandial glucose peaks at 45 min compared to the control (all adjusted *p* < 0.05). Peanut butter (PB) and peanut milk (PM) showed significantly lower response compared to WB at 30 min. The 4-h glucose iAUC revealed that PB exhibited a similar iAUC to WB. Cashew butter (CB) and cashew milk (CM) displayed iAUC values higher than WB, while whole peanuts (WP), whole cashews (WC), and PM showed lower iAUC values compared to WB. The 3-h glucose iAUC was highest for WB, CB and CM, whereas the 2-h glucose iAUC indicated that all nut forms were lower than the control WB. Peanuts, across all forms, yielded lower 2-h iAUC values compared to cashews (Figure 2).

**Figure 2:**
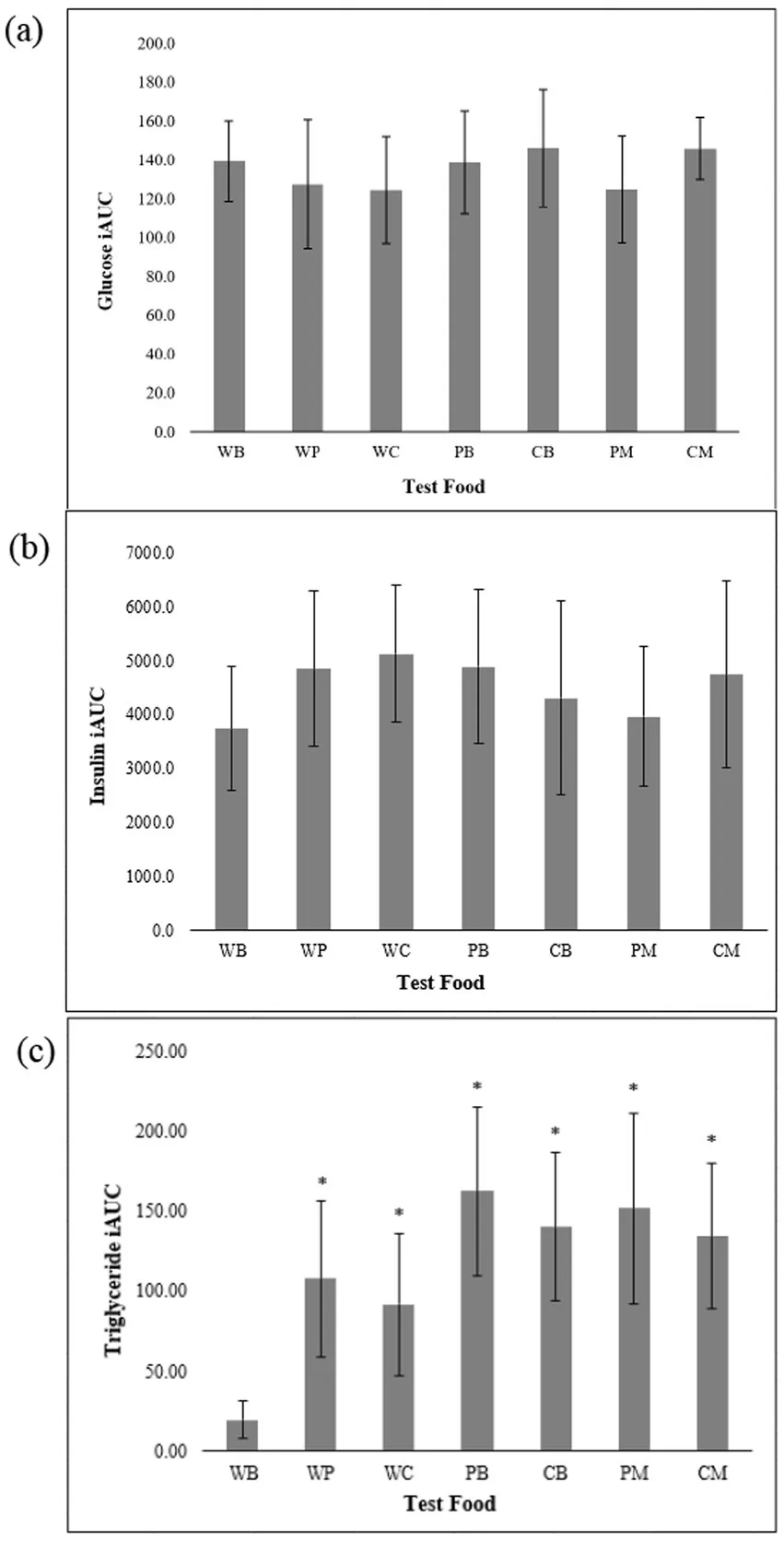
Mean **(a)** 4-h glycemic, **(b)** 4-h insulinemic and **(c)** 4-h triglyceride iAUC of white bread (control) and White Bread with Peanuts and Cashews in different forms, with 95% confidence intervals. The WB chart represents the mean of duplicate reference white bread sessions conducted for each participant. * Significant differences were observed between the 4-h triglyceride response iAUCs of WB and all treatments (*p* < 0.05). No statistically significant differences in iAUC were observed among the various nut treatment groups (*p* > 0.05).

#### 3.3.2 Insulin

WB elicited a rapid and substantial increase in insulin levels within the first 45 min, resulting in a high overall insulinemic response. WP, WC, and CM exhibited peak insulin levels comparable to WB. CB peaked at 30 min, while PB and PM peaked at 60 min. No significant differences were observed between the groups, for the food-time interaction, or in insulin peak values between each test food and the control (all adjusted *p* > 0.05). The nut forms demonstrated similar declines in insulin levels from the second hour onwards. Insulin levels returned to fasting baseline at 180 min for WB and at 240 min for the nut forms. All nut forms exhibited higher insulin iAUC values than the control. Among the nut forms, PM displayed the lowest iAUC, whereas CM showed the highest iAUC. No significant differences were observed between the groups (test food and control) for insulin iAUC (all adjusted *p* > 0.05).

#### 3.3.3 Triglycerides

Triglyceride levels for WB remained relatively stable throughout the 4-h period, whereas significant increases were observed in all test foods. Peak triglyceride levels occurred at 120 min for all nut forms (WC, PB, CB, PM, CM), except WP which peaked at 180 min. Triglyceride levels for the nut forms did not return to fasting baseline by 240 min. Compared to the control, significant increases in triglyceride peak values and iAUC were observed for all nut forms (*p* < 0.05). Among the test food, WC exhibited the lowest iAUC, while PB displayed the highest. Between nut forms, WP displayed significantly lower triglyceride levels at 120 and 180 min compared to PB. PB and PM exhibited 51 and 41% higher iAUC values, respectively, than WP, while CB and CM exhibited 53 and 46% higher iAUC values, respectively, than WC. Peanuts, across all forms, yielded a 15% higher iAUC than cashews.

### 3.4 Palatability rating

Palatability ratings for the test foods, measured using a 7-point hedonic scale, are detailed in Table 4. The control, white bread (WB), was rated at 4.4. Whole cashews (WC) and cashew butter (CB) were perceived as most palatable, with ratings of 4.8, whereas peanut milk (PM) received the lowest palatability rating of 4.2. No significant differences were observed in palatability ratings among the test foods (*p* > 0.05). Therefore, it is unlikely that variations in palatability influenced mastication patterns or subsequent digestion and absorption in this study.

**Table 4:** Palatability rating for the test foods.

Code	Test food	Palatability rating*
WB	White bread (control 1 and 2)	4.4 ± 1.3
WP	White bread + Whole peanuts	4.7 ± 1.0
PB	White bread + Peanut butter	4.5 ± 1.5
PM	White bread + Peanut milk	4.2 ± 1.3
WC	White bread + Whole cashews	4.8 ± 1.1
CB	White bread + Cashew butter	4.8 ± 1.3
CM	White bread + Cashew milk	4.5 ± 1.2

*Palatability rating: 1 to 7. 1: Not palatable, 7: Very palatable.

## 4 Discussion

This study examined the glycemic, insulinemic, and triglyceride responses to peanuts and cashews, consumed with white bread, in three forms: whole nuts, nut butter, and nut milk. The nuts attenuated glycemic responses while increasing insulinemic and triglyceride responses. Nut form significantly modulated triglyceride responses. Crucially, because this study evaluated realistic, household portions rather than strictly standardized test meals, the metabolic differences observed between the nut types and forms reflect the cumulative impact of their practical serving profiles rather than an isolated per-gram macronutrient comparison. These findings will be discussed in detail below.

### 4.1 Glycemic response

White bread (WB) elicited a rapid and substantial increase in glycemic response, followed by a decline in blood glucose levels below baseline after 2 hours, as illustrated in Figure 1. The 2-h glucose incremental area under the curve (iAUC) provided a more accurate representation of postprandial glycemic response compared to the 4-h iAUC, as it excluded the area below the fasting concentration. Both the 2-h glucose iAUC and the glycemic response curves demonstrated that the addition of nuts, in any form, significantly attenuated the glycemic response to white bread.

Peanuts and cashews possess a higher fat and protein content relative to white bread, which is predominantly carbohydrate. Previous studies have shown that the glycemic response of carbohydrate-rich meals can be reduced when consumed with fat or protein in both healthy and type 2 diabetic populations (18–21). This effect is attributed to mechanisms such as delayed gastric emptying (22) and enhanced insulin secretion via intestinal hormones known as incretins (23). Glucagon-like peptide 1 (GLP-1) and glucose-dependent insulinotropic polypeptide (GIP) are incretins secreted from the gastrointestinal tract following nutrient ingestion, stimulating glucose-dependent insulin secretion (24). Incretins promote pancreatic β-cell proliferation and insulin secretion, contributing to the maintenance of normal blood glucose levels (25). GLP-1 also regulates postprandial glucose by slowing gastric emptying and inhibiting glucagon secretion (24). Thus, the observed reduction in postprandial glycemic responses upon the addition of peanuts and cashews to white bread is likely due to the presence of fat and protein in the nuts.

Consuming nuts alone or with carbohydrates has been consistently shown to attenuate postprandial glucose responses (26). Numerous studies have examined the impact of nut consumption on glycemic control, reporting reductions in postprandial glucose responses across various populations, including healthy individuals, diabetic patients, and those with impaired glucose tolerance (4). Consuming nuts as an isolated preload blunts the glycemic response to a subsequent carbohydrate-dense meal via the ‘second-meal effect’, a process primarily driven by delayed gastric emptying, sustained gut hormone release (such as GLP-1), and attenuated free fatty acid rebound (27). A meta-analysis of 12 randomized controlled trials with follow-up periods exceeding 3 weeks concluded that tree nuts improve glycemic control in individuals with type 2 diabetes mellitus (T2DM) (28). While nut consumption reduces postprandial glucose, the effect is often small and may require large quantities of nuts (4).

Two acute dose–response studies, utilizing 0 g, 30 g, 60 g, and 90 g of almonds (29) and mixed nuts (26) with white bread, demonstrated that the 90 g dose significantly reduced glycemic response and glucose peak compared to the white bread control. Similarly, 85 g of pistachios (30) and 60 g of almonds (31) attenuated glycemic responses and blunted glucose peak height. This study, examining the effects of peanut and cashew forms on white bread, yielded consistent findings, demonstrating reductions in glycemic responses. The serving size of nuts in this study was 42.5 g, the standard USDA serving size. Despite being lower than the doses used in previous studies, significant reductions in glucose peaks for all nut forms were observed. Furthermore, peanut forms reduced glycemic response at higher levels compared to cashew forms, a finding that will be discussed further.

### 4.2 Insulinemic response

Peanuts and cashews elicited slightly higher insulin iAUC and insulinemic responses compared to white bread. This observation contrasted with the glucose results, where nut forms attenuated glycemic responses. While glycemic response is a significant predictor of insulin response, it accounts for only 23% of the variability, indicating the presence of other factors influencing insulin secretion beyond glucose elevation (32). This response can be attributed to the insulinotropic effects of the nut macronutrient matrix. Peanuts and cashews are rich in protein and unsaturated fatty acids, which are known to directly stimulate pancreatic β-cells via circulating amino acids and trigger the release of incretin hormones like GLP-1 and GIP. This allows for an increased insulin secretion without a concomitant increase in glucose concentrations (4, 24, 33–36). This study’s insulinemic response findings are also consistent with an acute study on pistachio consumption in individuals with metabolic syndrome (30).

Conversely, only one acute study in healthy participants reported a reduction in insulinemic response following almond consumption with white bread, though this reduction is not significant. It might be attributed to the delayed gastric emptying as the amount of protein and fat administered was substantially higher than in the present study (31). This would lead to a throttled gastric motility, and a slow, steady release of carbohydrates into the small intestine, which allows the pancreas to secrete insulin at a gradual, continuous rate. This ultimately results in a blunted, flattened peak height rather than an acute postprandial spike. A meta-analysis of 12 randomized controlled trials, conducted over periods exceeding 3 weeks, found no significant effect of nuts on fasting insulin or insulin sensitivity in T2DM (28). Similarly, 4-week nut supplementation studies with almonds in healthy adults (37) and pistachios in well-controlled T2DM patients (38) showed no impact on insulin sensitivity. Interestingly, 16- and 24-week almond trials demonstrated decreases in insulin and fasting insulin levels in subjects with prediabetes (39), and overweight or obese participants (40), respectively. A 4-month chronic pistachio consumption study also reported insulin-lowering effects in prediabetic individuals (41), suggesting that a reduction in insulinemic response may be observable after long-term nut consumption. Given the short-term nature of this study, no significant insulin-related effects were observed.

### 4.3 Triglyceride response

The emphasis on 4-h iAUC values in our primary lipid analysis reflects the physiological delay inherent to intestinal lipid absorption and chylomicron circulation, which require an extended postprandial monitoring window compared to the rapid 2-h clearance observed for glucose. The iAUC and triglyceride levels for white bread remained low throughout the 4-h period, consistent with its predominantly carbohydrate composition and low-fat content (1 g). Conversely, peanuts and cashews, which are rich in fat, led to a significant increase in triglyceride levels and iAUC, as expected when meal fat content is elevated (42). Triglyceride levels for the nut forms did not return to fasting baseline by 240 min. This does not necessarily indicate an adverse metabolic outcome, as prolonged lipid excursions may partly reflect the slower digestion and clearance of dietary fats from emulsified nut products (12). However, although acute postprandial lipemia is a normal physiological response to fat ingestion, repeated and prolonged postprandial hypertriglyceridemia has been associated with increased cardiovascular risk due to prolonged exposure to triglyceride-rich lipoproteins and their remnants (43). Therefore, interpretation of these acute findings should be considered alongside evidence from long-term studies demonstrating cardiovascular benefits associated with habitual nut consumption (44). Statistically significant differences in iAUC were observed between all nut forms and white bread. Furthermore, nut butter and nut milk exhibited 52 and 44% higher iAUC values, respectively, than whole nuts. A more detailed comparative analysis of nut types and forms will be presented in the subsequent section.

### 4.4 Significance between nuts and nut forms for glycemic, insulinemic and triglyceride response

In individuals with T2DM, the addition of almonds and peanuts to a low-carbohydrate diet has been shown to yield comparable improvements in postprandial blood glucose control (45). This study observed similar glycemic response reductions when peanuts and cashews were incorporated into a high-carbohydrate diet, specifically demonstrating lower glycemic responses for peanut forms during the initial 2 hours. This observed difference may be attributed to the contrasting macronutrient profiles of the nuts, as detailed in Table 2. Peanuts, with their lower carbohydrate (64%) and higher protein (37%) content compared to cashews, are expected to elicit a more gradual glycemic response. This aligns with existing research indicating that protein and fat can reduce glycemic responses in a dose-dependent manner, with protein exhibiting a greater effect than fat (21). The observed 15% higher triglyceride iAUC for peanuts compared to cashews may be due to the 1.7 g higher fat content in peanuts. Conversely, cashews, having a slightly higher sugar content and lower protein and fiber content relative to peanuts, are likely to result in a faster and higher spike in blood glucose levels (46). Regarding lipid response, the 15% higher triglyceride iAUC observed for peanuts compared to cashews may be explained by their 1.7 g higher fat content, despite both nuts being rich in monounsaturated fats. No significant differences in insulinemic responses were observed between the nut types. For triglyceride response, WP displayed significantly lower levels at 120 and 180 min compared to PB.

Nut form may influence gastric emptying and nutrient digestion, leading to variations in blood nutrient profiles and gut hormone responses (9, 47). Additionally, high lipid bioaccessibility has been suggested as a factor contributing to elevated postprandial responses (48). Previous acute studies examining the impact of whole nuts and nut butter in almonds (49), peanuts (48), and walnuts (50) reported no significant differences in glucose and insulin responses between these forms. This study, which included two nut types and nut milk as an additional form, similarly found no significant effects of nut type or form on glycemic and insulinemic responses. However, significant increases in triglyceride peak values and overall iAUC were observed for nut butter and nut milk compared to white bread, but not for whole nuts. Among the nut forms, whole nuts elicited the lowest triglyceride responses, followed by nut milk, with nut butter exhibiting the highest.

Lipid bioaccessibility is determined by the degree of cell wall rupture, which is influenced by grinding or mastication (51). The mechanical processing involved in converting whole nuts to nut butter, such as grinding, leads to cell wall disruption, increasing the availability of intracellular lipids and other nutrients for digestion and thus enhancing lipid bioaccessibility (47, 51). The digestibility of lipids in almonds has been shown to be closely related to meal structure and particle size distribution (52). Studies have indicated that whole nuts are not fully digested, resulting in a slower release of lipids during digestion (53, 54). Whole nuts, being solid, contain fat droplets up to four times larger than those in nut butter and nut milk (11). These larger fat droplets delay triglyceride hydrolysis and fatty acid release in the small intestine, leading to lower triglyceride responses (11). Conversely, the smaller fat droplets in nut butter and nut milk provide a larger surface area for gastric and pancreatic lipase binding, facilitating faster digestion (10). A study on the effect of food form and structure on lipid digestion and postprandial lipemic response reported a higher triglyceride iAUC following liquid food consumption compared to solid food over a six-hour period, indicating increased fat absorption from liquid food (11).

Contrary to this study’s hypothesis that nut milk would elicit the highest triglyceride response, nut butter was found to have the greatest effect. The study’s nut milk, prepared by blending with water, did not undergo the high-pressure and heat treatment used in commercial nut milk production, which disrupts the phospholipid and protein monolayers of nuts (13). The absence of heat treatment may have preserved the intact microstructure of oil bodies within the nut milk (55), resulting in lower lipid bioaccessibility and thus a lower triglyceride response compared to nut butter. Further research directly measuring of lipid bioaccessibility of different nut forms would be beneficial to elucidate the effect of commercial nut milk production versus non-commercial preparation.

Incorporating nuts into a high-carbohydrate diet may offer advantages for glycemic control. Notably, peanuts elicited lower glycemic responses compared to cashews. A notable finding in the present study was the elevated postprandial triglyceride response following the ingestion of processed nut forms (nut butter and nut milk) compared to whole nuts. While acute, transient increases in postprandial lipemia are a physiological consequence of enhanced lipid bioaccessibility and rapid emulsification, the long-term clinical implications warrant consideration. Chronic, sustained postprandial hypertriglyceridemia has been identified as an independent risk factor for cardiovascular disease due to the accumulation of atherogenic remnant lipoproteins (43). However, long-term epidemiological studies and clinical trials consistently demonstrate that habitual nut consumption regardless of form is associated with improved lipid profiles, reduced vascular inflammation, and lower cardiovascular mortality (44). This suggests that the transient postprandial triglyceride excursions observed with emulsified nut matrices may be offset by the chronic cardiovascular benefits of nut-derived unsaturated fatty acids, dietary fiber, and bioactive phytonutrients. Nevertheless, for populations at elevated cardiovascular risk, whole nuts may offer a preferred matrix by blunting both postprandial glycemic and lipemic spikes.

Furthermore, no significant effects of nut type or form were observed on acute insulinemic responses. Collectively, these findings suggest that for individuals aiming to manage postprandial blood glucose and lipid levels, prioritizing whole nuts over more highly processed forms like nut butter could be a beneficial dietary strategy.

This study had several limitations. The particle size of the nut forms was not measured, thus precluding the determination of size-related effects, including lipid bioaccessibility, which could also be assessed *in vitro*. Analysis of incretin hormones, specifically GLP-1 and GIP, was not conducted. Quantifying these hormones could have provided valuable mechanistic insights to support the explanations presented previously. Visual analog scales (VAS) were not assessed to correlate with hunger and satiety. Extending the study duration could further elucidate the long-term effects of nut forms on triglyceride levels, given that levels remained elevated after 4 hours. Replacing the white bread control with a high-fat meal could determine if nut forms have a triglyceride-lowering effect (56, 57). The generalizability of these findings is limited to individuals with normal BMI, as metabolic responses may differ in overweight or obese populations. Habitual bread or nut consumption among the participants were not assessed prior to the study. Although baseline fasting levels were standardized and participants served as their own controls within the cross-over design, differences in habitual bread or nut consumption could affect individual postprandial responses. Incorporating food frequency questionnaires in future research would help control for this factor. Test meals were not energy matched. The addition of peanuts or cashews substantially increased total energy, fat, and protein content compared with white bread alone. Consequently, observed effects should be interpreted as responses to realistic nut-containing meals rather than isolated effects of nut type or form itself. Additionally, due to the acute nature of this study, further long-term research is needed to fully understand the sustained effects of nut forms on glycemic, insulinemic, and triglyceride responses.

## 5 Conclusion

This study examined the glycemic, insulinemic, and triglyceride responses to white bread consumed with peanuts and cashews in three forms: whole nuts, nut butter, and nut milk. The addition of nuts attenuated glycemic responses, significantly reduced glucose peaks (*p* < 0.05), and slightly increased insulinemic responses compared to white bread alone. Peanut forms elicited lower glycemic responses compared to cashew forms, potentially due to their higher protein and lower carbohydrate content. Whole nuts elicited the lowest triglyceride responses among nut forms, while nut butter and nut milk exhibited higher triglyceride responses compared to whole nuts. Differences in protein and fat composition and lipid bioaccessibility among the nut types and forms may account for the observed variations in glycemic and triglyceride responses. In conclusion, the incorporation of peanuts and cashews into a high-carbohydrate meal (white bread) was found to improve glycemic control. While nut form significantly impacted postprandial triglyceride responses, long-term evidence supports the overall cardiovascular safety of nut intake. These findings suggest that prioritizing whole nuts over highly processed forms, such as nut butter and nut milk, may represent a potentially beneficial dietary strategy for individuals managing diabetes or cardiovascular disease risk.

## Data Availability

The original contributions presented in the study are included in the article/supplementary material, further inquiries can be directed to the corresponding author.

## Footnotes

1
Randomizer.org
